# Supplementary material for: A CRISPR mediated point‐of‐care assay for the detection of mucosal calprotectin in an animal model of ulcerative colitis
Source: Bioeng Transl Med. 2024 Sep 25;10(2):e10725. doi: 10.1002/btm2.10725 (PMC11883116; doi:10.1002/btm2.10725)
Supplement: Supplementary file 1 — DATA S1. Supporting information. [file BTM2-10-e10725-s001.docx]

**A CRISPR mediated point-of-care assay for the detection of mucosal calprotectin in an animal model of ulcerative colitis**

Selena Chia ^a, b^, Tianruo Guo ^a, b^, Ewa M. Goldys ^a^, Sophie C. Payne ^c, d^, Wenlu Duan ^a, b^, Nigel H. Lovell ^a, b^, Mohit N. Shivdasani ^a, b, #^, Fei Deng ^a, b, #, *^

a. Graduate School of Biomedical Engineering, UNSW Sydney, Sydney 2052, Australia

b. Tyree Foundation Institute of Health Engineering (iHealthE), UNSW Sydney, NSW 2052, Australia

c. Bionics Institute, Victoria 3002, Australia

d. Medical Bionics Department, University of Melbourne, Victoria 3010, Australia

* Correspondence: [fei.deng@unsw.edu.au](mailto:fei.deng@unsw.edu.au).

#These authors contributed equally to the work

**Supplementary Figures**


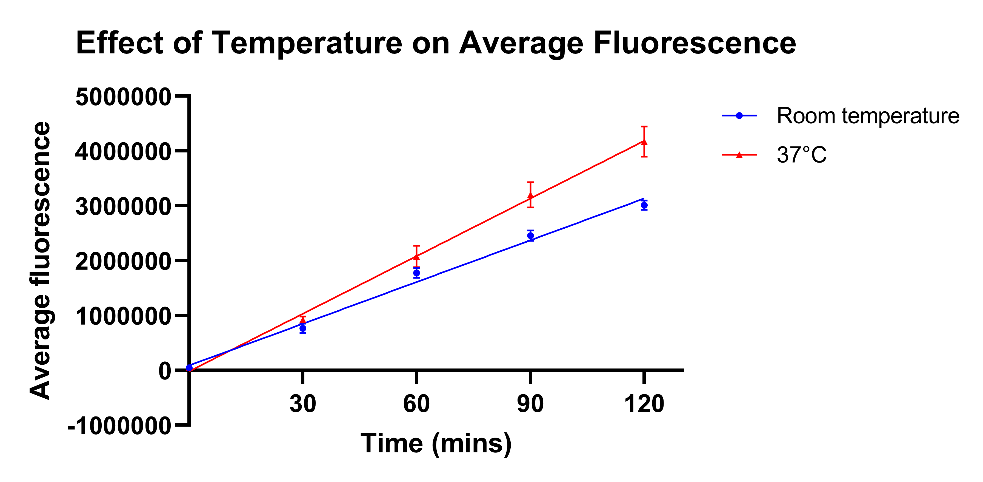


**Figure S1.** Fluorescence from CRISPR-target activity with 40 nM trigger ssDNA concentration over time in room temperature and 37°C temperature (n=3).

**Supplementary Table**

**Table S1**. Raw data for biosensor specificity (Figure 3B).

| Protein | Data 1 | Data 2 | Data 3 | Average | SD |
| --- | --- | --- | --- | --- | --- |
| calprotectin | 4486790 | 4256942 | 4339036 | 4360922.667 | 116476.5874 |
| IFN-y | 2204763 | 2592288 | 2424143 | 2407064.667 | 194326.1653 |
| IL-6 | 2191992 | 2292626 | 2428329 | 2304315.667 | 118601.3517 |
| IL-10 | 2320684 | 2245273 | 2527022 | 2364326.333 | 145856.4866 |
| TNF-a | 2448099 | 2566684 | 2545958 | 2520247 | 63335.55239 |
| IL-1b | 2611765 | 2505199 | 2471061 | 2529341.667 | 73393.15479 |
| BSA | 2416301 | 2319295 | 2500320 | 2411972 | 90590.10894 |
| Neg | 2391992 | 2292626 | 2428329 | 2370982.333 | 70248.70549 |
| Mixture | 4770447 | 4357815 | 4662028 | 4596763.333 | 213917.9735 |

**Table S2**. The recent state of art biosensors for detecting CP.

| **Method** | **LOD** | **Ref** |
| --- | --- | --- |
| Luminescent biosensor | 0.1 ng/µL | [1] |
| Molecularly imprinted photonic hydrogel | 0.007 ng/mL | [2] |
| CalproSmart | 30 µg/g | [3] |
| Electrochemical immunosensor | 137.7 fg/mL | [4] |
| Impedimetric aptasensor | 16.89 µg/g | [5] |
| Zn(II)-dependent DNAzyme | 9.89 nM | [6] |
| Calprotectin ligands | 15.6 ng/mL | [7] |
| Electrochemiluminescence emissive aptamer | 0.945 ng/mL | [8] |
| CRISPR assay | 1 ng/mL | This study |

**References**

1. Lan, T., et al., *Development of Luminescent Biosensors for Calprotectin.* ACS Chemical Biology, 2024.

2. Resende, S., et al., *Detection of serum calprotectin based on molecularly imprinted photonic hydrogels: A novel approach for IBD diagnosis.* Biosensors and Bioelectronics: X, 2023. **13**: p. 100313.

3. Vinding, K.K., et al., *Fecal calprotectin measured by patients at home using smartphones—a new clinical tool in monitoring patients with inflammatory bowel disease.* Inflammatory bowel diseases, 2016. **22**(2): p. 336-344.

4. Dong, L., et al., *An enzyme-free ultrasensitive electrochemical immunosensor for calprotectin detection based on PtNi nanoparticles functionalized 2D Cu-metal organic framework nanosheets.* Sensors and Actuators B: Chemical, 2020. **308**: p. 127687.

5. Aslan, Ç.G., et al., *Non‐invasive detection of Crohn's Disease with label‐free detection of calprotectin by impedimetric aptasensor.* Electroanalysis, 2023. **35**(12): p. e202300119.

6. Si, J., et al., *A Turn-Off Fluorescent Strategy for Calprotectin Detection Based on the Inhibitory Effect of Calprotectin upon the Activity of Zn (Ⅱ)-Dependent DNAzyme.* Chemosensors, 2022. **10**(12): p. 495.

7. Díaz-Perlas, C., et al., *High-affinity peptides developed against calprotectin and their application as synthetic ligands in diagnostic assays.* Nature Communications, 2023. **14**(1): p. 2774.

8. Lv, F., et al., *Amplification of an Electrochemiluminescence-Emissive Aptamer into DNA Nanotags for Sensitive Fecal Calprotectin Determination.* Analytical Chemistry, 2023. **95**(50): p. 18564-18571.
